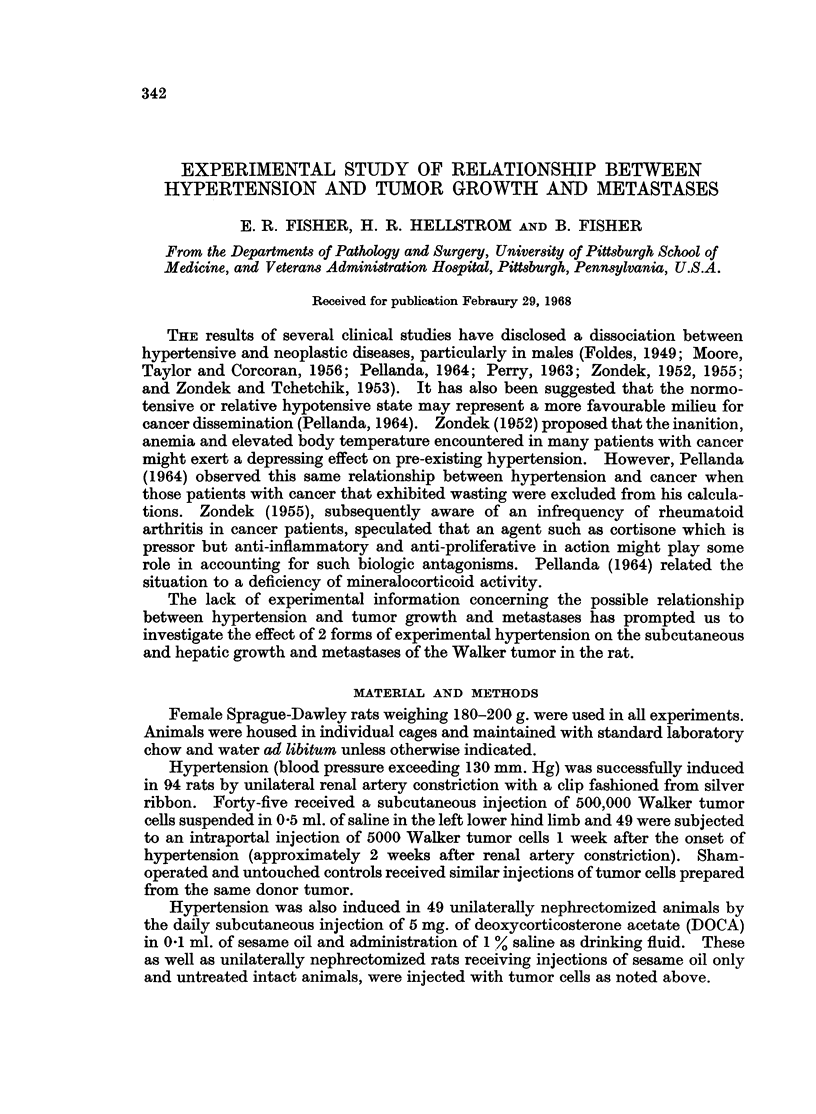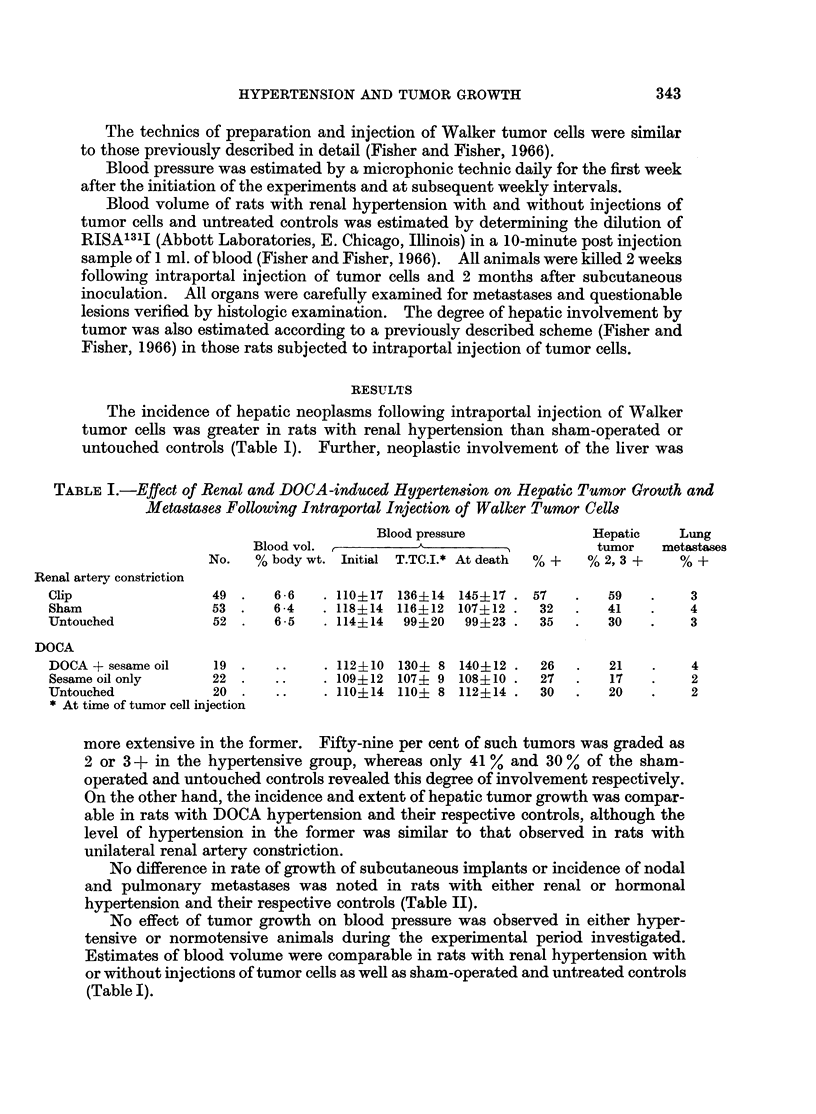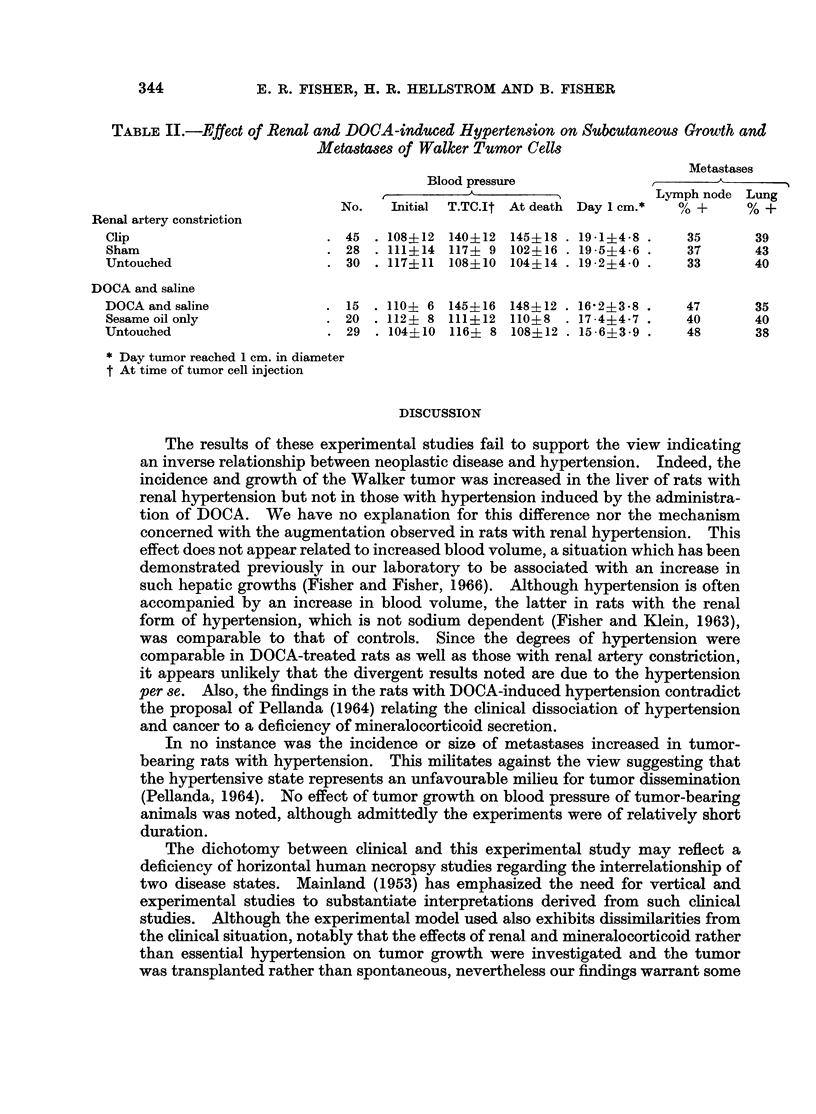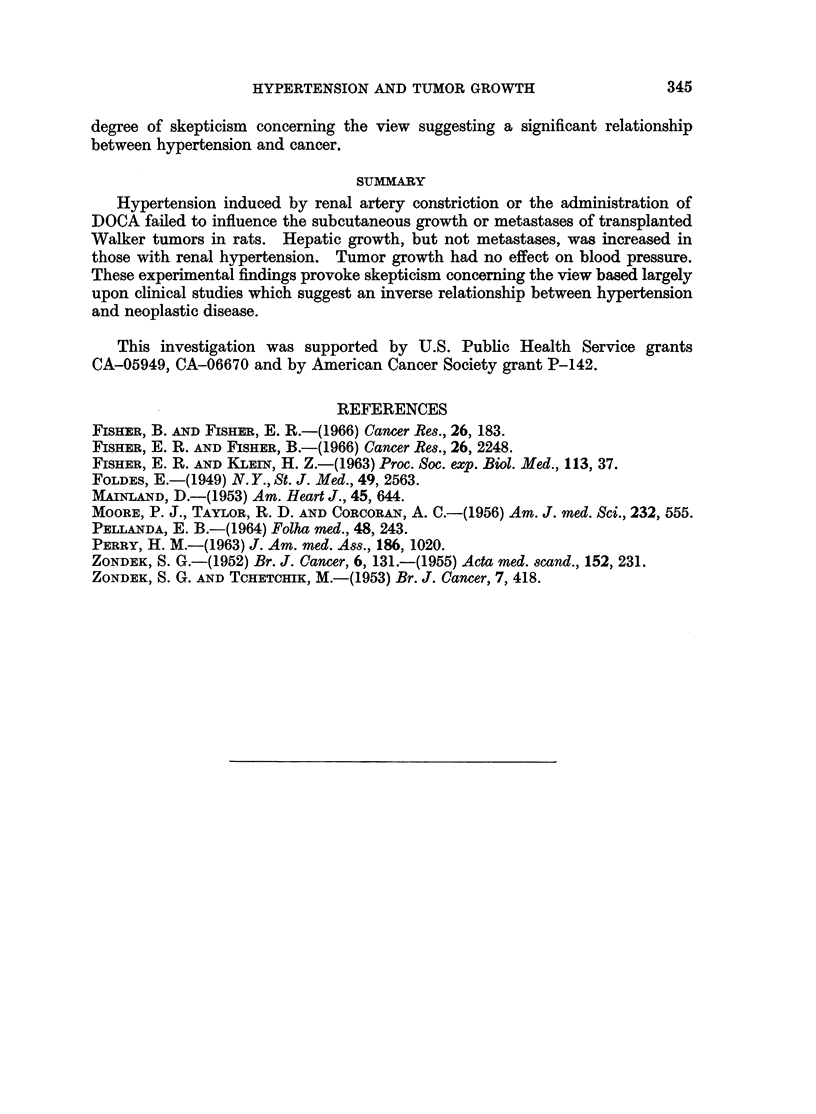# Experimental study of relationship between hypertension and tumor growth and metastases.

**DOI:** 10.1038/bjc.1968.42

**Published:** 1968-06

**Authors:** E. R. Fisher, H. R. Hellstrom, B. Fisher


### EXPERIMENTAL STUDY OF RELATIONSHIP BETWEEN HYPERTENSION AND TUMOR GROWTH AND METASTASES

E. R. FISHER, H. R. HELLSTROM AND B. FISHER

*From the Departments of Pathology and Surgery, University of Pittsburgh School of Medicine, and Veterans Administration Hospital, Pittsburgh, Pennsylvania, U.S.A.*

Received for publication Febraury 29, 1968

THE results of several clinical studies have disclosed a dissociation between hypertensive and neoplastic diseases, particularly in males (Foldes, 1949; Moore, Taylor and Corcoran, 1956; Pellanda, 1964; Perry, 1963; Zondek, 1952, 1955; and Zondek and Tchetchik, 1953). It has also been suggested that the normotensive or relative hypotensive state may represent a more favourable milieu for cancer dissemination (Pellanda, 1964). Zondek (1952) proposed that the inanition, anemia and elevated body temperature encountered in many patients with cancer might exert a depressing effect on pre-existing hypertension. However, Pellanda (1964) observed this same relationship between hypertension and cancer when those patients with cancer that exhibited wasting were excluded from his calculations. Zondek (1955), subsequently aware of an infrequency of rheumatoid arthritis in cancer patients, speculated that an agent such as cortisone which is pressor but anti-inflammatory and anti-proliferative in action might play some role in accounting for such biologic antagonisms. Pellanda (1964) related the situation to a deficiency of mineralocorticoid activity.

The lack of experimental information concerning the possible relationship between hypertension and tumor growth and metastases has prompted us to investigate the effect of 2 forms of experimental hypertension on the subcutaneous and hepatic growth and metastases of the Walker tumor in the rat.

### MATERIAL AND METHODS

Female Sprague-Dawley rats weighing 180–200 g. were used in all experiments. Animals were housed in individual cages and maintained with standard laboratory chow and water *ad libitum* unless otherwise indicated.

Hypertension (blood pressure exceeding 130 mm. Hg) was successfully induced in 94 rats by unilateral renal artery constriction with a clip fashioned from silver ribbon. Forty-five received a subcutaneous injection of 500,000 Walker tumor cells suspended in 0·5 ml. of saline in the left lower hind limb and 49 were subjected to an intraportal injection of 5000 Walker tumor cells 1 week after the onset of hypertension (approximately 2 weeks after renal artery constriction). Sham-operated and untouched controls received similar injections of tumor cells prepared from the same donor tumor.

Hypertension was also induced in 49 unilaterally nephrectomized animals by the daily subcutaneous injection of 5 mg. of deoxycorticosterone acetate (DOCA) in 0·1 ml. of sesame oil and administration of 1 % saline as drinking fluid. These as well as unilaterally nephrectomized rats receiving injections of sesame oil only and untreated intact animals, were injected with tumor cells as noted above.

The technics of preparation and injection of Walker tumor cells were similar to those previously described in detail (Fisher and Fisher, 1966).

Blood pressure was estimated by a microphonic technic daily for the first week after the initiation of the experiments and at subsequent weekly intervals.

Blood volume of rats with renal hypertension with and without injections of tumor cells and untreated controls was estimated by determining the dilution of RISA$^{131}$I (Abbott Laboratories, E. Chicago, Illinois) in a 10-minute post injection sample of 1 ml. of blood (Fisher and Fisher, 1966). All animals were killed 2 weeks following intraportal injection of tumor cells and 2 months after subcutaneous inoculation. All organs were carefully examined for metastases and questionable lesions verified by histologic examination. The degree of hepatic involvement by tumor was also estimated according to a previously described scheme (Fisher and Fisher, 1966) in those rats subjected to intraportal injection of tumor cells.

### RESULTS

The incidence of hepatic neoplasms following intraportal injection of Walker tumor cells was greater in rats with renal hypertension than sham-operated or untouched controls (Table I). Further, neoplastic involvement of the liver was

TABLE I.—*Effect of Renal and DOCA-induced Hypertension on Hepatic Tumor Growth and Metastases Following Intraportal Injection of Walker Tumor Cells*

| | No. | Blood vol. % body wt. | Blood pressure Initial | T.TC.I.* | At death | % + | Hepatic tumor % 2, 3 + | Lung metastases % + |
|---|---|---|---|---|---|---|---|---|
| Renal artery constriction | | | | | | | | |
| Clip | 49 | 6·6 | 110±17 | 136±14 | 145±17 | 57 | 59 | 3 |
| Sham | 53 | 6·4 | 118±14 | 116±12 | 107±12 | 32 | 41 | 4 |
| Untouched | 52 | 6·5 | 114±14 | 99±20 | 99±23 | 35 | 30 | 3 |
| DOCA | | | | | | | | |
| DOCA + sesame oil | 19 | .. | 112±10 | 130± 8 | 140±12 | 26 | 21 | 4 |
| Sesame oil only | 22 | .. | 109±12 | 107± 9 | 108±10 | 27 | 17 | 2 |
| Untouched | 20 | .. | 110±14 | 110± 8 | 112±14 | 30 | 20 | 2 |

* At time of tumor cell injection more extensive in the former. Fifty-nine per cent of such tumors was graded as 2 or 3+ in the hypertensive group, whereas only 41% and 30% of the sham-operated and untouched controls revealed this degree of involvement respectively. On the other hand, the incidence and extent of hepatic tumor growth was comparable in rats with DOCA hypertension and their respective controls, although the level of hypertension in the former was similar to that observed in rats with unilateral renal artery constriction.

No difference in rate of growth of subcutaneous implants or incidence of nodal and pulmonary metastases was noted in rats with either renal or hormonal hypertension and their respective controls (Table II).

No effect of tumor growth on blood pressure was observed in either hypertensive or normotensive animals during the experimental period investigated. Estimates of blood volume were comparable in rats with renal hypertension with or without injections of tumor cells as well as sham-operated and untreated controls (Table I).

TABLE II.—*Effect of Renal and DOCA-induced Hypertension on Subcutaneous Growth and Metastases of Walker Tumor Cells*

| | | Blood pressure | | | | Metastases | |
|---|---|---|---|---|---|---|---|
| | No. | Initial | T.TC.I† | At death | Day 1 cm.* | Lymph node % + | Lung % + |
| Renal artery constriction | | | | | | | |
| Clip | 45 | 108±12 | 140±12 | 145±18 | 19·1±4·8 | 35 | 39 |
| Sham | 28 | 111±14 | 117± 9 | 102±16 | 19·5±4·6 | 37 | 43 |
| Untouched | 30 | 117±11 | 108±10 | 104±14 | 19·2±4·0 | 33 | 40 |
| DOCA and saline | | | | | | | |
| DOCA and saline | 15 | 110± 6 | 145±16 | 148±12 | 16·2±3·8 | 47 | 35 |
| Sesame oil only | 20 | 112± 8 | 111±12 | 110±8 | 17·4±4·7 | 40 | 40 |
| Untouched | 29 | 104±10 | 116± 8 | 108±12 | 15·6±3·9 | 48 | 38 |

* Day tumor reached 1 cm. in diameter
† At time of tumor cell injection

### DISCUSSION

The results of these experimental studies fail to support the view indicating an inverse relationship between neoplastic disease and hypertension. Indeed, the incidence and growth of the Walker tumor was increased in the liver of rats with renal hypertension but not in those with hypertension induced by the administration of DOCA. We have no explanation for this difference nor the mechanism concerned with the augmentation observed in rats with renal hypertension. This effect does not appear related to increased blood volume, a situation which has been demonstrated previously in our laboratory to be associated with an increase in such hepatic growths (Fisher and Fisher, 1966). Although hypertension is often accompanied by an increase in blood volume, the latter in rats with the renal form of hypertension, which is not sodium dependent (Fisher and Klein, 1963), was comparable to that of controls. Since the degrees of hypertension were comparable in DOCA-treated rats as well as those with renal artery constriction, it appears unlikely that the divergent results noted are due to the hypertension *per se*. Also, the findings in the rats with DOCA-induced hypertension contradict the proposal of Pellanda (1964) relating the clinical dissociation of hypertension and cancer to a deficiency of mineralocorticoid secretion.

In no instance was the incidence or size of metastases increased in tumor-bearing rats with hypertension. This militates against the view suggesting that the hypertensive state represents an unfavourable milieu for tumor dissemination (Pellanda, 1964). No effect of tumor growth on blood pressure of tumor-bearing animals was noted, although admittedly the experiments were of relatively short duration.

The dichotomy between clinical and this experimental study may reflect a deficiency of horizontal human necropsy studies regarding the interrelationship of two disease states. Mainland (1953) has emphasized the need for vertical and experimental studies to substantiate interpretations derived from such clinical studies. Although the experimental model used also exhibits dissimilarities from the clinical situation, notably that the effects of renal and mineralocorticoid rather than essential hypertension on tumor growth were investigated and the tumor was transplanted rather than spontaneous, nevertheless our findings warrant some degree of skepticism concerning the view suggesting a significant relationship between hypertension and cancer.

SUMMARY

Hypertension induced by renal artery constriction or the administration of DOCA failed to influence the subcutaneous growth or metastases of transplanted Walker tumors in rats. Hepatic growth, but not metastases, was increased in those with renal hypertension. Tumor growth had no effect on blood pressure. These experimental findings provoke skepticism concerning the view based largely upon clinical studies which suggest an inverse relationship between hypertension and neoplastic disease.

This investigation was supported by U.S. Public Health Service grants CA-05949, CA-06670 and by American Cancer Society grant P-142.

### REFERENCES

Fisher, B. and Fisher, E. R.—(1966) *Cancer Res.*, **26**, 183.
Fisher, E. R. and Fisher, B.—(1966) *Cancer Res.*, **26**, 2248.
Fisher, E. R. and Klein, H. Z.—(1963) *Proc. Soc. exp. Biol. Med.*, **113**, 37.
Foldes, E.—(1949) *N.Y., St. J. Med.*, **49**, 2563.
Mainland, D.—(1953) *Am. Heart J.*, **45**, 644.
Moore, P. J., Taylor, R. D. and Corcoran, A. C.—(1956) *Am. J. med. Sci.*, **232**, 555.
Pellanda, E. B.—(1964) *Folha med.*, **48**, 243.
Perry, H. M.—(1963) *J. Am. med. Ass.*, **186**, 1020.
Zondek, S. G.—(1952) *Br. J. Cancer*, **6**, 131.—(1955) *Acta med. scand.*, **152**, 231.
Zondek, S. G. and Tchetchik, M.—(1953) *Br. J. Cancer*, **7**, 418.